# Gut-Ex-Vivo system as a model to study gluten response in celiac disease

**DOI:** 10.1038/s41420-021-00430-2

**Published:** 2021-03-12

**Authors:** Mara Gagliardi, Nausicaa Clemente, Romina Monzani, Luca Fusaro, Eleonora Ferrari, Valentina Saverio, Giovanna Grieco, Elżbieta Pańczyszyn, Flavia Carton, Claudio Santoro, Sara Del Mare-Roumani, Sivan Amidror, Nissan Yissachar, Francesca Boccafoschi, Silvia Zucchelli, Marco Corazzari

**Affiliations:** 1 Department of Health Science, University of Piemonte Orientale, Novara, Italy; 2 Center for Translational Research on Autoimmune and Allergic Disease (CAAD), University of Piemonte Orientale, Novara, Italy; 3 Interdisciplinary Research Center of Autoimmune Diseases (IRCAD), University of Piemonte Orientale, Novara, Italy; 4 The Mina and Everard Goodman Faculty of Life Sciences, Bar-Ilan Institute of Nanotechnology and Advanced Materials, Bar-Ilan University, Ramat-Gan, Israel

**Keywords:** Coeliac disease, Tissue engineering

## Abstract

Celiac disease (CD) is a complex immune-mediated chronic disease characterized by a consistent inflammation of the gastrointestinal tract induced by gluten intake in genetically predisposed individuals. Although initiated by the interaction between digestion-derived gliadin, a gluten component, peptides, and the intestinal epithelium, the disorder is highly complex and involving other components of the intestine, such as the immune system. Therefore, conventional model systems, mainly based on two- or three-dimension cell cultures and co-cultures, cannot fully recapitulate such a complex disease. The development of mouse models has facilitated the study of different interacting cell types involved in the disorder, together with the impact of environmental factors. However, such in vivo models are often expensive and time consuming. Here we propose an organ ex vivo culture (gut-ex-vivo system) based on small intestines from gluten-sensitive mice cultivated in a dynamic condition, able to fully recapitulate the biochemical and morphological features of the mouse model exposed to gliadin (4 weeks), in 16 h. Indeed, upon gliadin exposure, we observed: i) a down-regulation of cystic fibrosis transmembrane regulator (CFTR) and an up-regulation of transglutaminase 2 (TG2) at both mRNA and protein levels; ii) increased intestinal permeability associated with deregulated tight junction protein expression; iii) induction and production of pro-inflammatory cytokines such as interleukin (IL)-15, IL-17 and interferon gamma (IFNγ); and iv) consistent alteration of intestinal epithelium/villi morphology. Altogether, these data indicate that the proposed model can be efficiently used to study the pathogenesis of CD, test new or repurposed molecules to accelerate the search for new treatments, and to study the impact of the microbiome and derived metabolites, in a time- and cost- effective manner.

## Introduction

Celiac disease (CD) is a chronic immune-mediated disease unleashed by gliadin, a gluten component, intake in predisposed individuals carrying Human Leukocyte Antigen (HLA) DQ2/DQ8 (ref. ^1^). CD pathogenesis is characterized by the activation of a cascade of signaling pathways culminating in a chronic pro-inflammatory condition and damage of the intestinal barrier. The imbalanced intestinal barrier permeability, activation of CD4 T cells, and production/release of pro-inflammatory cytokines are induced by the interaction of gliadin peptides with the chemokine CXC motif receptor 3 (CXCR3), thus promoting tight junction (TJ) disassembly and gliadin crossing the intestinal epithelium barrier^2,3^. Very recently, a link between Cystic Fibrosis syndrome and CD has been described, in which Cystic Fibrosis Transmembrane Regulator (CFTR) represents a key factor^4^. Indeed, gliadin-derived active peptides generated by digestion are able to bind CFTR, thus inhibiting its physiological activity, and resulting in up-regulation/activation of Transglutaminase 2 (TG2) and Nuclear Factor Kappa-light-chain-enhancer of activated B cells (NF-kB). This, in turn, increases the production and release of pro-inflammatory cytokines^4^. However, although CD primarily affects the small intestine, patients can also suffer from extra-intestinal manifestations, thus increasing the complexity of the disease^5,6^.

The only available treatment for patients affected by CD is a lifelong adherence to a gluten-free diet (GFD), which impacts on quality of life and cannot totally prevent accidental exposure to gluten, due to residual gluten content from food transformation or food cross contaminations.

Therefore, experimental systems are required to model CD and better recapitulate the onset and progression of this pathology, to better understand the molecular mechanisms underlying its pathogenesis and to design and develop new therapeutic approaches.

Although human intestinal biopsies are currently used, and mouse models of the disease have been developed and intensively used to study CD^7–9^, the development of ex vivo systems might importantly: (i) increase the overall amount of tissue to study, (ii) reduce the invasiveness, (iii) reduce the overall time to perform experiments, (iv) decrease experimental costs, and (v) positively impact on ethical issues.

Here we discuss the use of a gut-ex-vivo system (GEVS) to cultivate small intestine organs from gluten-sensitive mice (GS), able to recapitulate the pathogenesis of CD observed in the whole animals extensively exposed to gluten (4 weeks)^4^, after only 16 h exposure to active gliadin peptides. Indeed, we demonstrated a consistent down-regulation of CFTR and up-regulation of TG, a compromised intestinal permeability evidenced by deregulated expression of TJ proteins, the production of pro-inflammatory cytokines at both mRNA and protein levels, and intestinal epithelium damage, perfectly overlapping with the above-mentioned mouse model.

## Results

### Basic features of a GEVS derived from gluten-sensitive mice

**Fig. 1:**
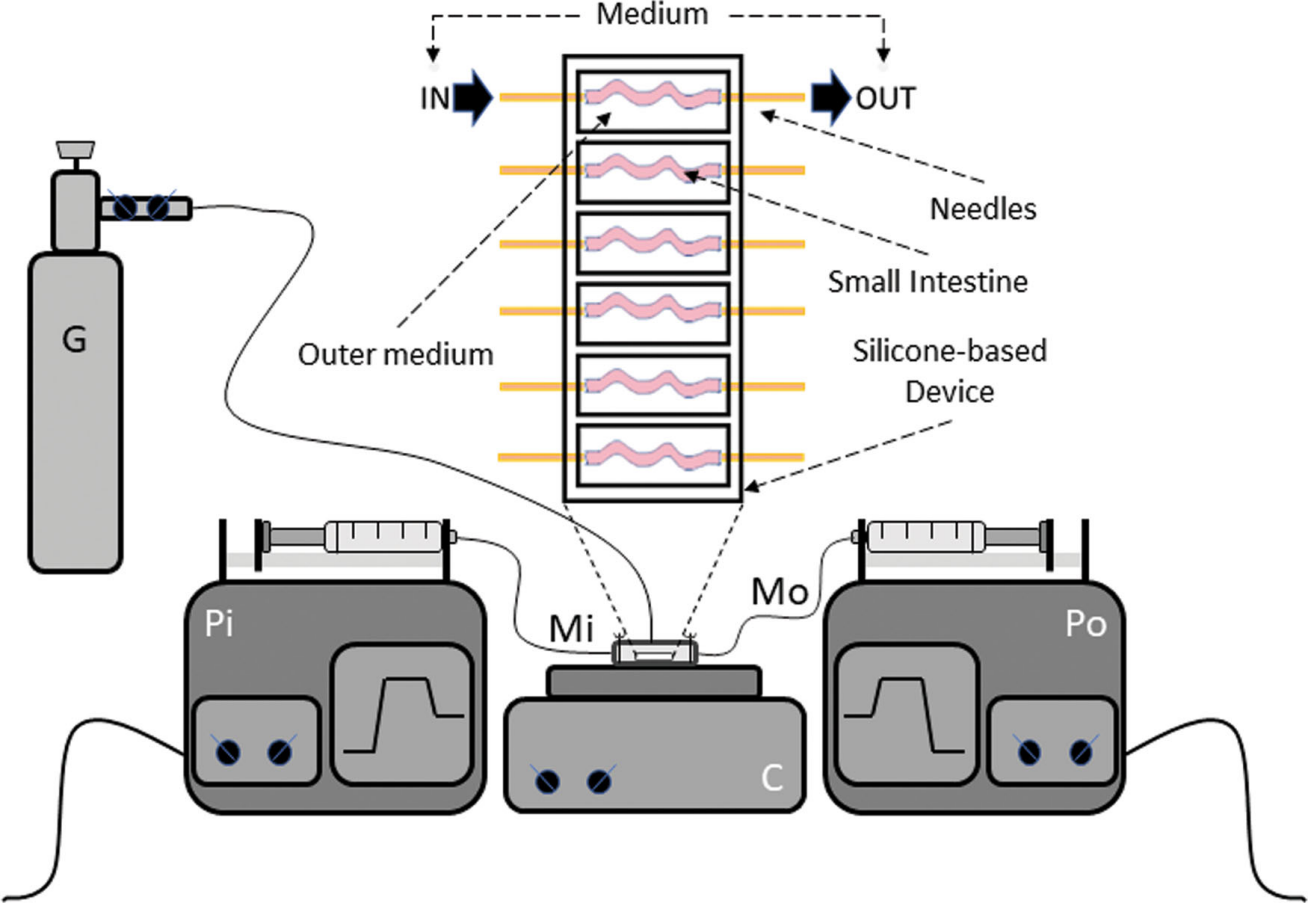
The GEVS. Schematic representation and silicone-based device consisting of 6 independent chambers in which small intestines (s.i.) are inserted and connected to two needles allowing the flow of complete medium in the inner intestinal compartment (luminal flow). Each chamber is also filled with a complete medium, in which s.i. are immerged, to sustain the full viability of tissues. The circuit allowing the flow of the medium through each s.i. consists of two synchronized pumps, which inject (Pi) and suck (Po) the culture medium. The temperature is set to 37 °C and maintained by a heating plate; proper oxygenation is allowed by humidified air/O_2_/CO_2_ gas mixture blown into the device chamber. Pi infusing pump; Po sucking pump; Mi medium entering; Mo medium exit; G air/O_2_/CO_2_ cylinder.

An ex vivo organ system to culture small intestines was developed by using the protocol previously developed by Yissachar and colleagues^10^. To this aim, small intestines from 13-day-old gluten-sensitive mice were surgically resected (2–3 cm) and inserted into a silicone-based device. Each basic unit (chamber) of the device has a paired input and output, which is connected to the lumen of intestine, allowing the introduction of a complete medium with additional treatments. Each unit is also filled with sterile complete medium to support complete viability of the cultured organ. Oxygenation and pH control of the outer medium is supported by an air/O_2_/CO_2_ gas mix blown into the closed chamber. The complete device is composed of six chambers for parallel culturing. Two multiplexed syringe pumps are connected to each chamber (intestine) to regulate inputs and outputs. A heating block is used to maintain physiological temperature of the whole culture (37 °C; Fig. 1). The outer intestinal environment of each chamber can also be connected to multiplexed syringe pumps to enable the introduction and complete replenishment of medium, if necessary (Fig. 1). All experiments were performed by culturing freshly explanted small intestines for 16 h.

### Tissues cultivated in the GEVS are viable and sensitive to ER stress induction, in a dose-dependent manner

**Fig. 2:**
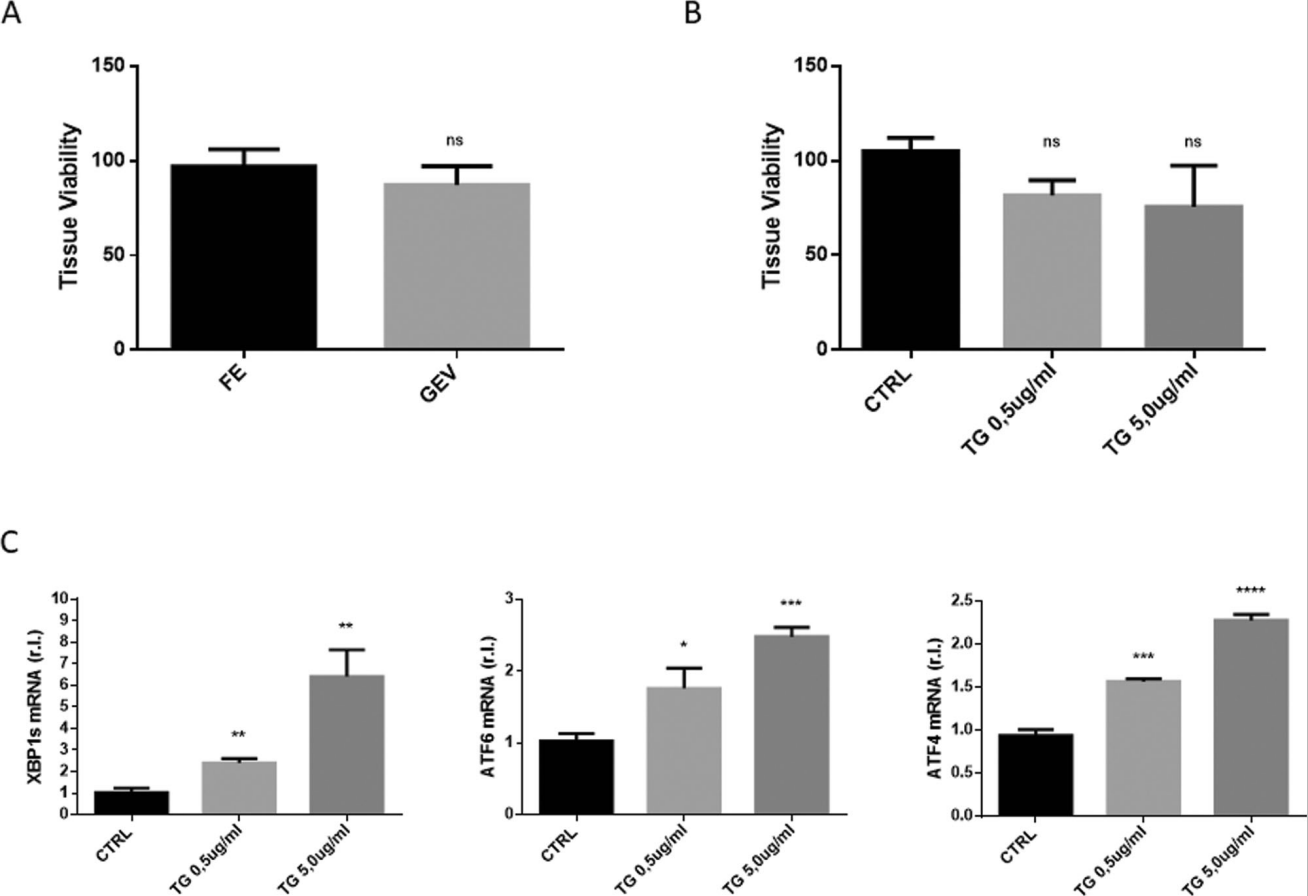
A viable and tunable system. Small intestines (s.i.) from Balb/c mice were cultured 16 h in the gut-ex-vivo (GEV) system described in Fig. 1, and tissue viability was evaluated by MTT assay and compared to freshly explanted (FE) matched controls (**A**; *n* = 3). S.i. were cultured 16 h in the gut-ex-vivo system, unexposed (CTRL) or exposed to TG 0.5 or 5.0 ug/ml, in complete medium, and tissue viability (**B**; *n* = 3) was evaluated by MTT assay or UPR (unfolded protein response) induction (**C**) was evaluated by qRT-PCR. Experiments were performed in triplicate and repeated three times. Data are represented as ‘fold over control’, r.l. relative levels. Histograms represent mean ± SD; ns non-significant; * *p* < 0.05; ** *p* < 0.01; *** *p* < 0.001; **** *p* < 0.0001.

To check the ability of the reconstituted whole GEVS to maintain complete viability of tissue, freshly explanted small intestines from 13-day-old Balb/c mice were cultivated in static complete medium (1 ml/chamber) and infused (luminal flow; 100 μl/h) with complete medium. Tissue viability was evaluated after 16 h and compared with freshly explanted small intestines (controls), by MTT assay. Data reported in Fig. 2A clearly show that organs maintained for 16 h in the GEVS didn’t show significant differences in terms of viability with respect to control.

To check whether tissues cultivated with the GEVS are also prone to efficiently respond to appropriate stimuli, we analyzed a signaling pathway routinely studied in our laboratory, such as endoplasmic reticulum stress (ER Stress). To this aim, small intestines from Balb/c mice were cultured for 16 h and infused with complete medium alone (CTRL) or supplemented with a well-known ER stress inducer such as Thapsigargin (TG) at 0.5 or 5.0 μg/ml^11,12^. Tissue viability was evaluated by MTT assay and data reported in Fig. 2B indicate that all tissues were viable and that TG treatments did not significantly alter tissue viability. Tissues were then homogenized and total RNA was extracted. Real-time quantitative reverse transcription PCR (qRT-PCR) analysis was carried out to evaluate the expression of well characterized ER stress markers such as X-box binding protein 1 (XBP1), activating transcription factor 6 (ATF6) and ATF4. Data reported in Fig. 2C clearly show that TG treatment resulted in a full and a dose-dependent ER stress induction, as evidenced by enhanced up-regulation of indicated ER stress markers.

These data indicate that cultured small intestines were viable and sensitive to ER stress induction, in a dose-dependent manner.

### Small intestines cultivated in the GEVS are prone to induce the early markers of CD by stimulation with gliadin-derived peptides

**Fig. 3:**
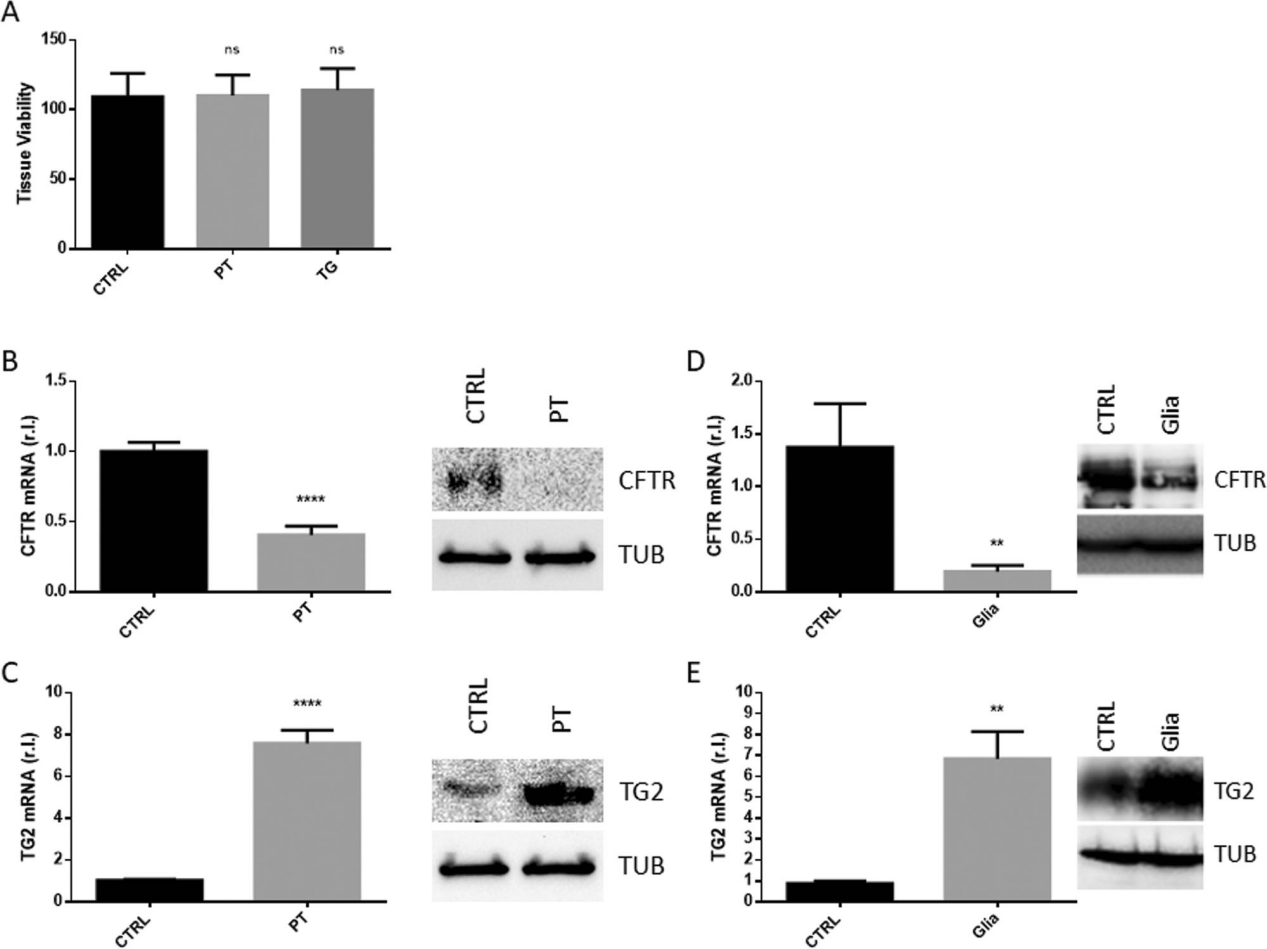
Early CD markers. S.i. from GS (gluten-sensitive) mice were explanted, cultured 16 h in presence of PT (peptic tryptic digest Gliadin; 2 mg/ml), TG (5.0 ug/ml), or untreated (CTRL), and cell viability (**A**; n = 3), was evaluated by MTT assay. Next, CFTR (**B**), or TG2 (**C**) expression levels were evaluated in the same experimental condition as in A by qRT-PCR and western blotting analysis. The expression levels of both CFTR (**D**) and TG2 (**E**) were also evaluated in GS (gluten-sensitive) mice unexposed or exposed 4 weeks to gliadin (Glia), at both mRNA and protein levels. Experiments were performed in triplicate and repeated three times. Data are represented as ‘fold over control’, r.l. relative levels. histograms represent mean ± SD; ns non-significant; * *p* < 0.05; ** *p* < 0.01; *** *p* < 0.001; **** *p* < 0.0001. Western blotting analysis is representative of three independent experiments.

To verify the hypothesis by which the GEVS could be used as a model to study human CD pathogenesis, the small intestine from gluten-sensitive mice was used^4^. To this aim, tissues were cultured 16 h in complete medium and CD was induced by infusing (luminal flow) complete medium containing 2 mg/ml of a tryptic digestion of gliadin (PT), containing the active peptides of gliadin, known to stimulate CD in this mouse model^4^. Complete medium (CTRL) or supplemented with 5.0 μg/ml TG were used as controls. Figure 3A shows that tissues exposed to PT were as viable as controls. Next, the early stages of CD were evaluated by measuring the level of the gliadin peptide target CFTR. As reported in Fig. 3B, tissues exposed to PT were characterized by consistent down-regulation of CFTR compared to control, at both mRNA and protein level. Then, we evaluated the expression of the key enzyme involved in the pathogenesis of CD, TG2, in the same experimental conditions. Interestingly, we observed a substantial enhanced expression of TG2 at both mRNA and protein levels (Fig. 3C). Importantly, the revealed down-regulation of CFTR and the up-regulation of TG2 observed in tissues cultured in the GEVS and exposed to PT were similar to those observed in the well characterized gluten-sensitive (GS) mouse model, in which mice were exposed to gliadin for 4 weeks, by oral gavage (Fig. 3D)^4^.

### Gliadin-derived peptides dysregulate intestinal barrier permeability in GEVS cultivated tissues

**Fig. 4:**
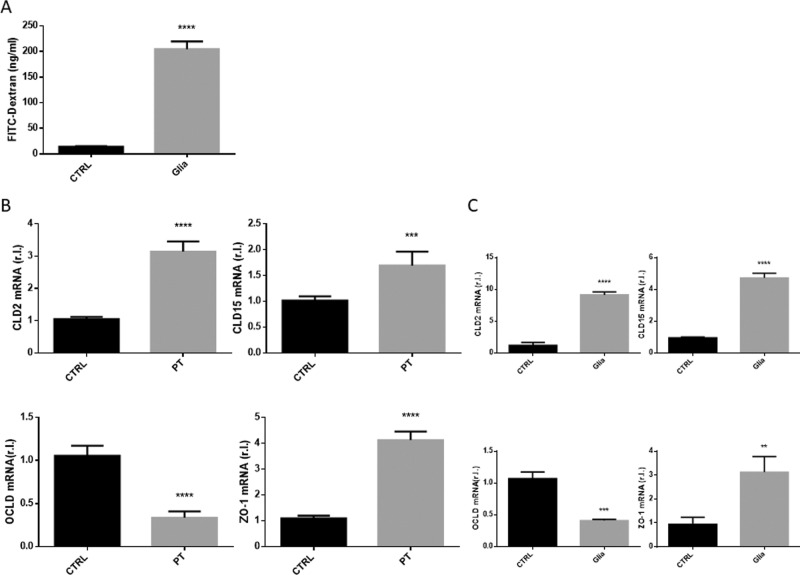
Intestinal barrier permeability. S.i. permeability was evaluated in mice unexposed or exposed 4 weeks to gliadin (Glia), by means of FITC-Dextran released in the plasma (**A**). The expression of the tight junction proteins claudin 2 (CLD2), claudin 15 (CLD15), occludin (OCLD), or ZO-1 were evaluated in s.i. from GS (gluten-sensitive) mice were explanted, cultured 16 h in presence of PT (peptic tryptic digest Gliadin; 2 mg/ml), or untreated (CTRL), by qRT-PCR (**B**). The expression levels (mRNA) of the same markers were also evaluated in s.i. of GS mice unexposed (CTRL) or exposed (Glia) 4 weeks to gliadin (**C**). Experiments were performed in triplicate and repeated three times. Data are represented as ‘fold over control’, r.l. = relative levels. Histograms represent mean ± SD; ns non-significant; * *p* < 0.05; ** *p* < 0.01; *** *p* < 0.001; **** *p* < 0.0001.

CD pathogenesis is characterized by progressive gliadin-stimulated impaired intestinal barrier permeability, due to TJs disassembly. The altered permeability can be evaluated in the whole CD mouse model previously described, by measuring the gliadin-stimulated release of FITC-Dextran from the intestinal lumen into the mouse plasma (Fig. 4A) or the altered expression of TJ proteins such as claudin 2 (CLD2) and 15 (CLD15), occludin (OCLD) or zonula occludens-1 (ZO-1)^4^. Indeed, our analysis revealed an enhanced expression of CLD2, CLD15 and ZO-1, and a down-regulation of OCLD (Fig. 4B) with an extent perfectly overlapping those from the whole GS mouse model reported in Fig. 3C, evaluated by qRT-PCR.

These data indicate that the intestinal permeability impairment associated with CD pathogenesis and recapitulated in the CD mouse model exposed to gliadin is also fully evidenced in GS-derived small intestines cultivated in the GEVS and exposed 16 h to PT.

### Inflammation was efficiently stimulated in tissues cultivated in the GEVS by stimulation with gliadin-derived peptides

**Fig. 5:**
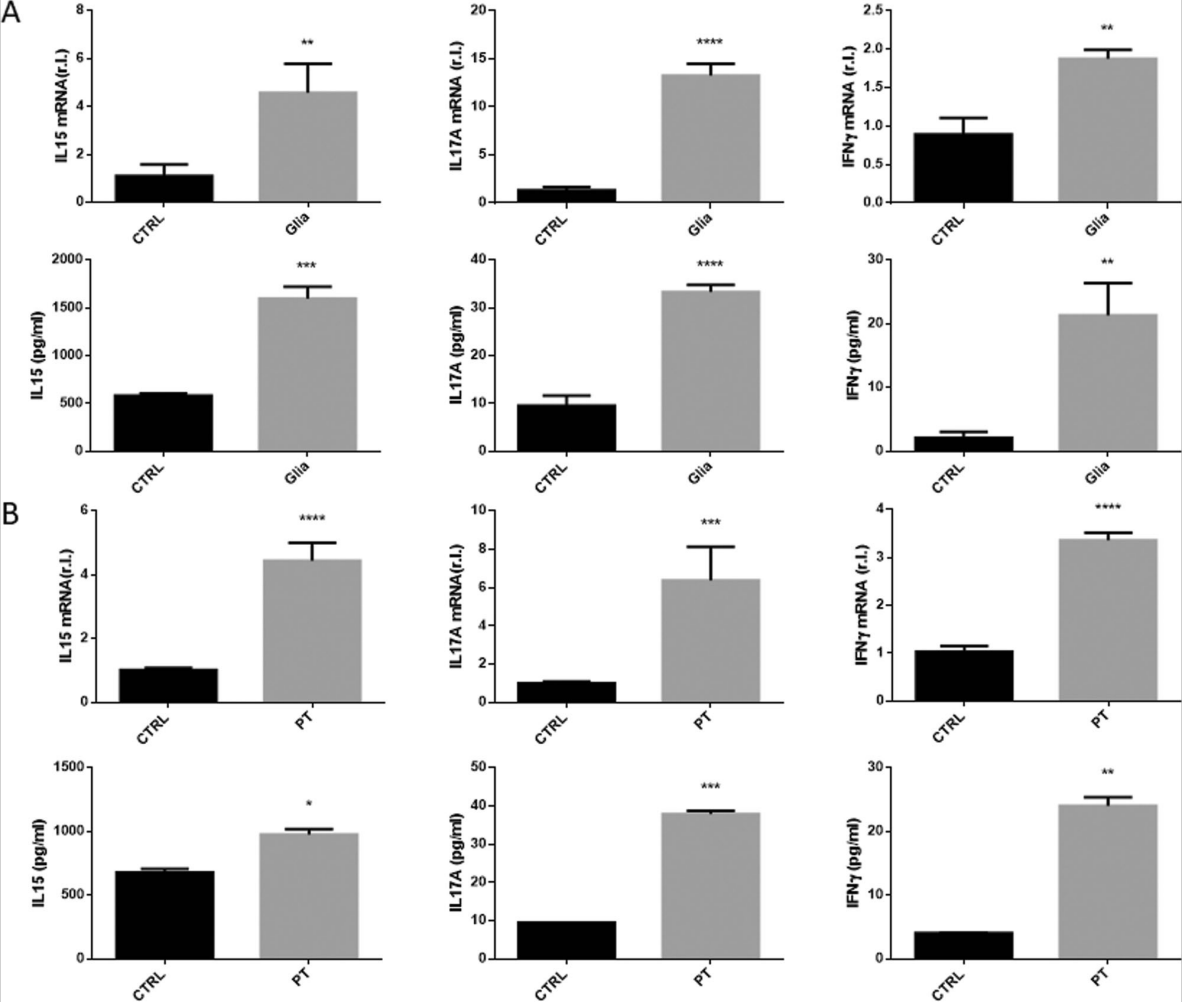
Pro-inflammatory response. S.i. were treated as described in Fig. 3 and the production/release of pro-inflammatory cytokines such as IL-15, IL-17, and IFNγ were evaluated at both mRNA (**A**, upper panels) or protein (**A**, bottom panels) levels by qRT-PCR analysis or ELISA, respectively, as indicated. **B** The same analysis was also carried out in s.i. lysates from gluten-sensitive mice unexposed (CTRL) or exposed (Glia) 4 weeks to gliadin. Experiments were performed in triplicate and repeated three times. mRNA expression levels are represented as ‘fold over control’, r.l. relative levels. Histograms represent mean ± SD; ns non-significant; * *p* < 0.05; ** *p* < 0.01; *** *p* < 0.001; **** *p* < 0.0001.

Altered permeability of the intestinal barrier together with decreased expression of CFTR and increased expression/activity of TG2 observed in patients affected by CD upon gluten consumption is followed by inflammation and immune system activation, finally resulting in autoimmune disease onset. Thus, gliadin exposure of GS mice results in active intestinal production of pro-inflammatory cytokines such interleukin (IL)-15, IL-17 and interferon gamma (IFNγ), as shown in Fig. 5A (ref. ^4^). Therefore, to verify whether a similar pro-inflammatory scenario can also be replicated in our organ cultures, small intestines from GS mice were cultured 16 h in the absence (CTRL) or presence (PT) of PT (2 mg/ml) in the infusing medium and total RNA or proteins were extracted. The expression of the above-mentioned pro-inflammatory mediators was then evaluated by qRT-PCR, while their secretion was evaluated by ELISA. Interestingly, data reported in Fig. 5A clearly show a consistent increased expression of the three genes (Fig. 5A, upper panels) paralleled by enhanced production of the cytokines (Fig. 5A, bottom panels), with respect to controls.

Thus, these data indicate that a pro-inflammatory response is actively mounted in tissues cultivated in the GEVS and exposed to gliadin peptides, reproducing with good approximation what happens in GS mice exposed to gliadin (Fig. 5B).

### Intestinal villi morphology was compromised by gliadin-derived peptides in tissue cultivated in the GEVS

**Fig. 6:**
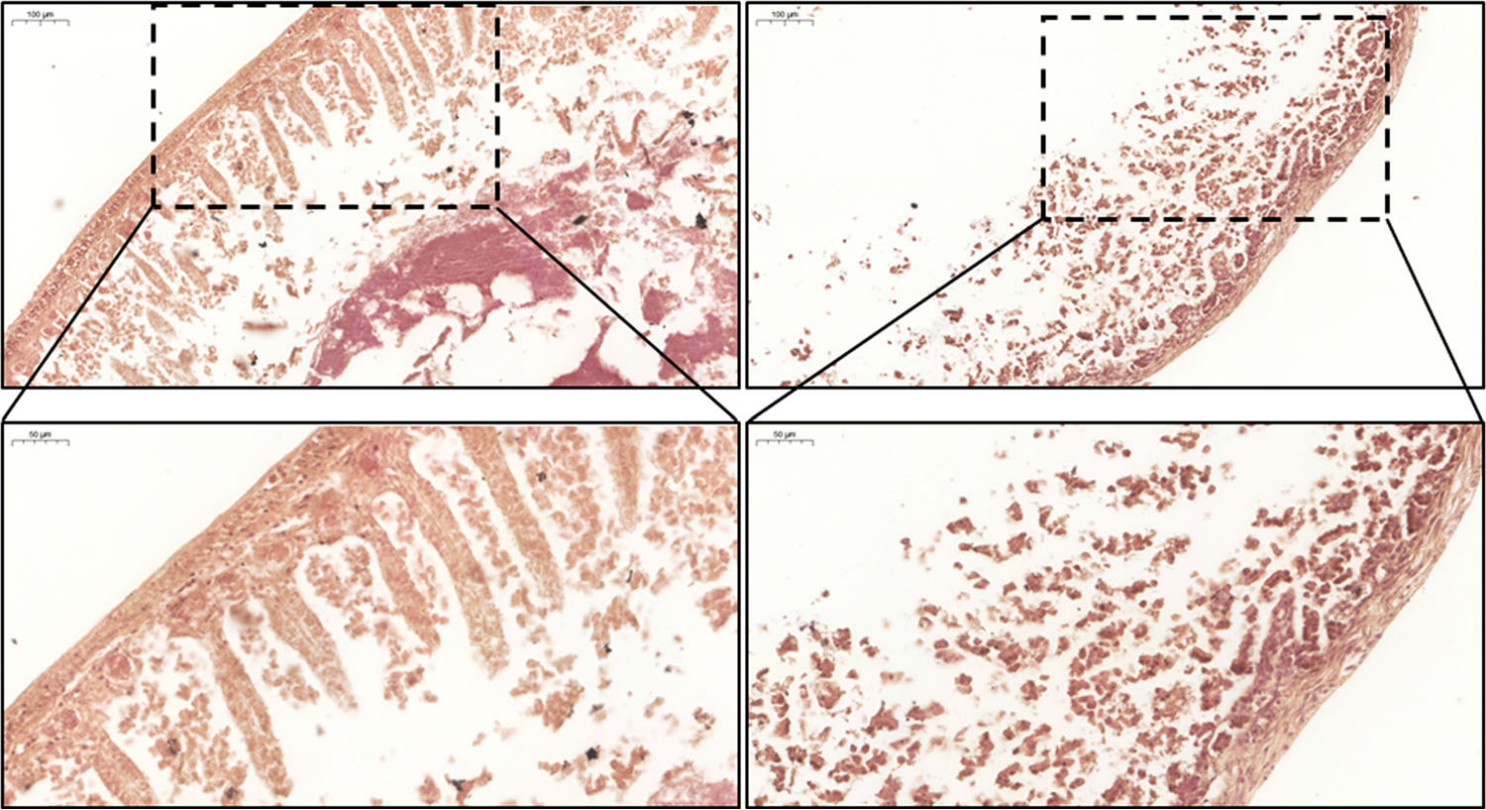
Small intestine morphology. S.i. from GS (gluten-sensitive) mice were cultured 16 h in the gut-ex-vivo system in the presence (PT; upper right panels) or absence (CTRL; upper left panels) of active gliadin peptides, and tissue morphology was evaluated in sections stained with H&E (magnification =×10; bar = 100 μm). A magnification of selected areas is reported in the bottom panels (magnification = ×20; bar = 50 μm).

Gluten intake from predisposed individuals also results in intestinal barrier tissue damage such as villi morphology alterations and atrophy, and immune cell infiltration. These typical CD features can also be evidenced in GS mice exposed to gliadin^4^. To verify whether a similar feature can also be reproduced in an ex vivo organ culture, we performed a hematoxylin and eosin (H/E) staining of small intestines cultivated 16 h in the absence (CTRL) or presence (PT) of gliadin-derived peptides, in the infusing medium. As shown in Fig. 6, gliadin peptides exposure resulted in a clear and deep alteration of tissue morphology, compared to control. In fact, while the muscular layer was quite preserved, a significant change involved mucosal and submucosal layers, leading to a complete absence of intestinal villi.

Therefore, our data show that 16 h exposure to PT is sufficient to induce a change in the intestinal morphology typical of CD pathogenesis, in a gut-ex-vivo culture of small intestines from GS mice.

## Discussion

Although it is not a lethal disease, CD is a worldwide problem for which there is still no effective therapeutic treatment. However, it significantly affects the health and sociability of the individual if it is not promptly diagnosed. Early diagnosis is therefore also essential to prevent complications that could be irreversible, such as growth retardation, osteoporosis, and abnormal dentition, in childhood^13^. Importantly several studies also suggest that delaying and gradually introducing gluten in the diet can reduce the risk of CD development in childhood^14^.

To date, lifelong adherence to a GFD is the only option for patients suffering from this disease, which however could reduce the quality of life and sometimes does not translate into complete remission.

Active research on the molecular mechanisms underlying the induction and progression of the pathology, together with the development of effective therapeutic treatments, is limited by the current biological in vitro models, which mainly consist of patient’s biopsies and/or cell culture/co-culture systems. Indeed, although the use of biopsies could represent the best model due to the direct derivation from affected patients, its invasive nature limits the amount of the biological sample, which is also affected by very limited proliferative capacity. On the other hand, primary or immortalized cells, such as Caco-2 (a human colorectal adenocarcinoma cells) and THP-1 (a human monocytic cell line derived from an acute monocytic leukemia patient), can be used to study the intestinal barrier features and/or the interaction between cells of the tissue barrier (epithelium and immune system) but cannot recapitulate the complexity of CD. Therefore, more complex models are urgently required. Of note, several mouse models have been developed and used in the research which can recapitulate the human pathology, such as gluten-sensitive (GS) Balb/c mice^7^, humanized mice expressing HLA-DQ2/DQ8 (ref. ^9^) or NOD mice^8^. However, these models also are time consuming and expensive. For example, GS mice must be exposed for at least four weeks to gliadin peptides, administered orally 2/3 times/week (by gavage)^4^.

Here we propose an organ ex vivo system (gut-ex-vivo) that efficiently reduces the experimental duration and is cost effective. The system, based on that previously developed by Yissachar and colleagues^10^, makes it possible to cultivate the intestines of 13-day-old GS mice and to stimulate a response to gliadin that is completely superimposable to the mouse model (GS exposed to gliadin peptides for 4 weeks), in just 16 h of stimulation. Indeed, we found a consistent CFTR down- and TG2 up-regulation at both mRNA and protein levels, paralleled by an altered intestinal barrier permeability and a pro-inflammatory response, evidenced by pro-inflammatory cytokine expression. Finally, we also found an altered morphology of the epithelium, associated with villi damage/atrophy.

Collectively these results indicate that the proposed GEVS recapitulates the intestinal physiology and can be efficiently used to study the development of human CD. Furthermore, it can be potentially and effectively used to test new molecules to accelerate the search for new treatments, as well as drug repositioning, and also to study the impact of the microbiome and derived metabolites, in a time- and cost-effective manner.

We are aware that the proposed intestinal ex vivo culture has many intrinsic limitations, such as, among others, the study of extra-intestinal damage associated with gliadin exposure and the gut-neuroimmune axis, at least in prolonged stimulation that mimics the condition of the chronic disease. However, we think the proposed model can efficiently be used to dissect the early stages of gliadin exposure, responsible for CD onset and progression, at the molecular level.

## Material and methods

### Mice and treatments

Balb/c mice were obtained from Envigo (Envigo, Huntingdon, UK). 8 weeks old of three-generation gluten-free mice (Mucedola S.r.l., Milan, Italy) were challenged with a GFD for all along with the time of the experiments (CTRL; *n* = 3), or challenged via oral gavage with gliadin (Sigma-Aldrich, Saint Louis, MO, USA, 5 mg/daily for 1 week, then 5 mg/daily thrice a week for 3 weeks) for 4 weeks (*n* = 3) (ref. ^4^). At the end of the last daily treatment mice were sacrificed and the intestine and the blood were collected and used for the analysis described below.

All procedures were approved by the local Ethics Committee for Animal Welfare (IACUC No 849) and conformed to the European Community regulations for animal use in research (2010/63 UE).

### Silicone-based device and organ culture

The silicone-based device was prepared in accordance to the procedure described by Yissachar N. and colleagues with minor modification^10^.

The custom-fabricated fluidic chip holds up to six individual gut tissue fragments in an isolated chamber. Each chamber is 8 mm wide and 25 mm long to accommodate mouse gut fragments of varying sizes and to provide a nutrient supply. The nutrient-rich environment is created by sealing the fluidic chip with a lid to maintain an O_2_/CO_2_-rich atmosphere and to prevent outside contaminations. Both the chip and the lid are made from poly(dimethylsiloxane) (PDMS; Sylgard 184 Elastomer base; Sigma-Aldrich) by replica molding of a 3D printed master (produced by Yissachar’s Laboratory). The masters are baked overnight in an oven at 90 °C to fully cure the material. Each master consists of a base and a frame. PDMS base and cross-linker are mixed together in a 10:1 ratio and poured into the mold. The PDMS is de-gassed in a vacuum chamber for 30 min to remove air bubbles, then the filled molds are placed in the oven overnight at 55 °C until the PDMS is fully cured. After gently removing the solid PDMS plates from the mold, they are subsequently glued to a glass slide (75 mm x 50 mm; Corning, NY, USA) using universal sealant (100% silicone sealant) and cured for a night at room temperature. These slides form the top and bottom of the device. Two fluidic connections are made to each chamber (one input, one output) at the appropriate locations with a needle (18 G needles that link the pump to the device, in input and in output). The area around each needle is also sealed with silicone sealant and left to cure overnight. The surgical thread, which will be used to secure the gut segments in silicone-based device, is knotted around the luminal input and output ports. The entire system is sterilized by dry-cycle autoclaving at 121 °C for 20 min.

To control the flow inside the lumen for each chamber on the device, the input and output ports are connected to a set of two multiplex syringe pumps capable of infusion and aspiration of fluid from six syringes in parallel^10^, with minor modification. Thus, the first pump is loaded with six syringes, each for every gut fragment (small intestine), and flows the lumen content into the input ports. The second pump (with six syringes respectively) drains these inputs.

During the experiment, the temperature of the device is maintained at 37 °C using a standard lab hot plate and a heat spreader was made and placed on the hot plate to ensure optimal heat transfer. A mixture of 5% CO_2_ and 95% O_2_ is provided to the device from a compressed gas cylinder connected to a regulator. This is important to maintain pH of the culture medium. The gases are humidified using standard oxygen humidifier; the flow from the regulator is connected to the device lid through a 14 G flat-end needle and an additional 20 G needle was added in the opposite side of the lid for pressure release^10^. Prior to dissection, input/output ports were flushed using sterile culture medium and gut fragment (small intestine) was gently flushed in sterile medium and fixed over the luminal input and output ports using the pre-positioned surgical thread. After inserting each intestinal fragment into the chamber, the device is placed on the heat conducting adapter and sealed using the previously manufactured lid.

### Tissue cultures and treatments

Each intestine section was infused with serum-free tissue culture medium containing Iscove’s Modified Dulbecco’s Medium (IMDM, Gibco, CA, USA) supplemented with 20% KnockOut serum replacement (Gibco), 2% B-27 and 1% of N-2 supplements (Gibco), 1% L-glutamine, 1% non-essential amino acids (NEAA), 1% HEPES and stimulated with Thapsigargin (0.5 or 5.0 µg/ml, T9033 Sigma-Aldrich) or PT-Gliadin (peptic tryptic digest Gliadin; 2.5 mg/ml, Sigma-Aldrich). The tissue culture medium was loaded into 5 ml syringe (for short-term experiments, 16 h) and infused into the device input ports by a syringe pump (flow rate of 99ul/h) (as described by Yissachar N. et al. with minor modification).

Gliadin from wheat (Sigma-Aldrich) was digested, as previously described^15^. In detail, each 50 g gliadin (G-3375, Sigma-Aldrich) was firstly dissolved in 500 mL 0.2 N HCl for two hours at 37 °C with 1 g pepsin (Sigma-Aldrich). The resultant peptic digest was further digested by the addition of 1 g trypsin (Sigma-Aldrich), after pH adjusted to 7.4 using 2 N NaOH; next, the solution was incubated at 37 °C for four hours with a vigorous agitation. Finally, the mixture was boiled to inactivate enzymes for 30 min and was stored at −20 °C (hereafter referred as PT-gliadin).

### Tissue viability assay

Tissue viability was assessed through MTS assay (CellTiter 96^®^ AQueous Non-Radioactive Cell Proliferation Assay, Promega, Milan, Italy). Samples were placed in a 48 well plate and cultured for 4 h with Dulbecco’s Modified Eagle’s Medium (DMEM – Euroclone S.p.a., Milan, Italy) enriched with 10% fetal bovine serum (FBS), 2 mM glutamine, 100 U/ml Penicillin, 0.1 mg/ml Streptomycin (Euroclone) and 3-(4,5-dimethylthiazol-2-yl)-5-(3-carboxymethoxyphenyl)-2-(4-sulfophenyl)-2H-tetrazolium solution following the kit instructions. After the incubation, absorbance of 100 µl of medium solution from each sample was measured by UV-VIS spectrophotometry (Victor X4, PerkinElmer, Buckinghamshire, UK), at a wavelength of 490 nm. Measures were proportional to cell viability.

### qRT-PCR

Trizol reagent (Invitrogen, Burlington, ON, Canada) was used to isolate total RNA, as indicated by the supplier. The AMV Reverse Transcriptase kit (Promega) was used to generate cDNA following the manufacturer’s recommendations. Quantitative PCR reactions were performed by using the CFX96 thermocycler (Bio-Rad Laboratories, CA, USA). Supplementary Table 1 shows the primers sequence for all amplicons, designed by using the online IDT PrimerQuest Tool software (IDT, Integrated DNA Technologies Inc., IA, USA; https://eu.idtdna.com/Primerquest/Home/Index). Results were normalized by using mouse GAPDH as internal control^11^.

### Western blotting analysis

The whole small intestine lysates were obtained by using the Cell Lytic buffer (Sigma-Aldrich) supplemented with a protease inhibitors cocktail (Sigma-Aldrich) plus phosphatases inhibitors (Na_3_VO_4_ 1 mM; NaF 10 mM), and resolved by electrophoresis through SDS-PAGE, and electroblotted onto nitrocellulose (Protran, Sigma-Aldrich) membranes. Membranes were incubated with indicated primary antibodies in 5% non-fat dry milk (Bio-Rad) in PBS plus 0.1% Tween20 overnight at 4 °C. Primary antibodies were: anti-CFTR (clone CF3, ab278, Abcam, Cambridge, UK) 1:500, anti-TG2 (clone CUB7402, NeoMarkers, Invitrogen) 1:500, and anti-tubulin (Santa Cruz, CA, USA) 1:5000. Detection was achieved using horseradish peroxidase-conjugate secondary antibody (1:5000; Jackson ImmunoResearch; Cambridge, UK) and visualized with ECL plus (Amersham Biosciences Corp., Little Chalfont, UK). Images were acquired by using a ChemiDoc™ Touch Imaging System (Bio-Rad) and analysed by Image Lab software (Bio-Rad), as previously described^16^.

### ELISA

IL-15, IL-17A, and IFNγ were measured in small intestine lysates by using the mouse IL-15 DuoSet ELISA, the mouse IL-17 Quantikine ELISA Kit, or the mouse IFNγ Quantikine ELISA Kit (Bio-Techne, MN, USA), as recommended by the supplier. ODs were analysed by a SPARK Multimode Microplate Reader (Tecan). Values were normalized to total protein concentration evaluated by Bradford analysis, as previously reported^4^.

### Hematoxylin/Eosin staining

For hematoxylin/eosin staining, samples were fixed in 4% formaldehyde solution, dehydrated and paraffin embedded. Samples were then cut into sections of 5 μm, rehydrated and soaked in hematoxylin (Sigma-Aldrich) for 15 min, and in eosin solution (0.05% eosin, Sigma-Aldrich, in distilled water and acetic acid) for 1 min. Finally, samples were dehydrated, mounted with Q Path® Coverquick 2000 (VWR International Ltd, PA, USA) and observed with an optic microscope (Leica DM2500, Leica, Wetzlar, Germany). Samples images have been acquired through a Leica DFC7000T camera (Leica) and analyzed with Leica Application Suite X software (Leica).

### Statistical analysis

All experiments were performed at least in triplicate and statistical analysis was performed using GraphPad software (GraphPad Software; GraphPad Prism 6). Student’s *t* test was used to determine statistical significance. A *p*-value of equal to or less than 0.05 was considered significant. Experiments were performed in triplicate and repeated at least three times. mRNA expression levels were represented as ‘fold over control’, r.l. relative levels. Histograms represent mean ± SD; ns non-significant; * *p* < 0.05; ** *p* < 0.01; *** *p* < 0.001; **** *p* < 0.0001.

## Supplementary information

Supplementary Table1
