# Supplementary material for: Gut-Ex-Vivo system as a model to study gluten response in celiac disease
Source: Cell Death Discov. 2021 Mar 12;7:45. doi: 10.1038/s41420-021-00430-2 (PMC7955131; doi:10.1038/s41420-021-00430-2)
Supplement: Supplementary file 1 — Supplementary Table1 [file 41420_2021_430_MOESM1_ESM.pdf]

Supplementary Table1. Primers sequence.

Primer	Sequence
IL-15_F	CAGCAAGGACCATGAAGA
IL-15_R	GGCTGAGTTCCACATCTAAC
IL-17a_F	CGCAATGAAGACCCTGATAG
IL-17_R	CTTGCTGGATGAGAACAGAA
INF$_\gamma$_F	CCACATCTATGCCACTTGAG
INF$_\gamma$_R	CTCTTCCTCATGGCTGTTTC
ATF4_F	GTTTAGAGCTAGGCAGTGAAG
ATF4_R	CCTTTACACATGGAGGGATTAG
ATF6_F	GATGGTGACAACCAGAAAGA
ATF6_R	TGGAGGTGGAGGCATATAA
XBP1s_F	AGTCCGCAGCAGGTG
XBP1s_R	GGTCCAACTTGTCCAGAATG
TG2_F	AAGAGCGAAGGGACATACT
TG2_R	TGAGCACAGACCCATCTT
CFRT_F	AGGAGGACAGGGATGATAAG
CFTR_R	GTAGACACACCAGGAGTCTG
CLD15_F	GGGACCCTCCACATACTT
CLD15_R	CATACTTGGTTCCAGCATACA
CLD2_F	CCTCGCTGGCTTGTATTATC
CLD2_R	AAAGACTCCACCCACTACA
OCLN_F	TCTTTGGAGGAAGCCTAAAC
OCLN_R	CTGCTCTTGGGTCTGTATATC
ZO-1_F	ACCAGACATACCCTCCTTAC
ZO-1_R	GAGATGAGGCTTCTGCTTTC
GAPDH_F	TTCAACGGCACAGTCAAG
GAPDH_R	CCAGTAGACTCCACGACATA

***Note**: m = mouse; h = human; XBP1s_F/R = primers designed to amplify the spliced (mature) mRNA of XBP1.*
